# Ricolinostat (ACY-1215) suppresses proliferation and promotes apoptosis in esophageal squamous cell carcinoma via miR-30d/PI3K/AKT/mTOR and ERK pathways

**DOI:** 10.1038/s41419-018-0788-2

**Published:** 2018-07-26

**Authors:** Jinlin Cao, Wang Lv, Luming Wang, Jinming Xu, Ping Yuan, Sha Huang, Zhehao He, Jian Hu

**Affiliations:** Department of Thoracic surgery, The first Affiliated Hospital, School of Medicine, Zhejiang University, 79 Qingchun Road, Hangzhou, 310003 China

## Abstract

Ricolinostat (ACY-1215), a first-in-class selective HDAC6 inhibitor, exhibits antitumor effects alone or in combination with other drugs in various cancers. However, its efficacy in esophageal cancer remains unclear. In this study, we found that the high expression of HDAC6 was associated with poor prognosis in esophageal squamous cell carcinoma (ESCC) tissues. Then, we identified that ACY-1215 significantly inhibited cellular proliferation in ESCC, and caused G2/M phase arrest and apoptosis. We further demonstrated that ACY-1215 treatment reduced the expression of PI3K, P-AKT, P-mTOR, and P-ERK1/2 and increased that of Ac-H3K9 and Ac-H4K8. In addition, using miRNA microarray and bioinformatics analysis, we detected that ACY-1215 promoted miR-30d expression, and PI3K regulatory subunit 2 (PIK3R2) was a direct target of miR-30d. Anti-miR-30d partially rescued the G2/M phase arrest and apoptosis caused by ACY-1215 treatment. The reductions in PI3K, P-AKT, and P-mTOR expression were also partially reversed by miR-30d inhibitor. Furthermore, the effects of ACY-1215 inhibited ESCC proliferation were validated in a mouse xenograft model in vivo. In conclusion, our study showed that ACY-1215 suppressed proliferation and promoted apoptosis in ESCC via miR-30d/PI3K/AKT/mTOR and ERK pathways and that ACY-1215 may be a promising antitumor agent in ESCC.

## Introduction

Esophageal cancer is one of the most aggressive malignancies worldwide^1^. The two main histopathological types of esophageal cancer are squamous cell carcinoma and adenocarcinoma. In the highest-risk areas, 90% of cases are esophageal squamous cell carcinoma (ESCC)^1,2^. Despite advances in medical technology, such as early screening, and surgery combined with chemotherapy or radiotherapy, the prognosis of esophageal cancer is still very poor^3,4^. Recently, targeted therapy has undergone unparalleled development in various cancers; however, only trastuzumab (targeting HER2) and ramucirumab (VEGFR-2 inhibitor) have so far been recommended by the National Comprehensive Cancer Network for patients with esophageal cancer^5^. Therefore, elucidation of the underlying pathogenesis of esophageal cancer and identification of effective biological agents are urgently needed.

HDAC6 (histone deacetylase 6) is a unique isoenzyme with two deacetylase domains, and belongs to the class IIb histone deacetylase family^6^. This enzyme is involved in multiple functions through deacetylase-dependent or deacetylase-independent mechanisms regulating many physiological or pathological processes^7^. Therefore, extensive efforts have been devoted to the search for selective HDAC6 inhibitors. Some inhibitors have exhibited extreme selectivity for HDAC6, and have shown broad application prospects in the treatment of cancer^8^.

Ricolinostat (ACY-1215) is a first-in-class potent and selective HDAC6 inhibitor^6,8,9^. It has demonstrated antitumor effects alone or in combination with other drugs in various cancers, such as multiple myeloma, lymphoma, glioblastoma, melanoma, and inflammatory breast cancers^10–15^. ACY-1215 activates potent acetylation of α-tubulin and multiple stress-related mechanisms to induce tumor cell apoptosis^9,10^. Several clinical trials have tested the efficacy of ACY-1215 in malignancies, either as a single treatment or in combination with other agents^6,9^. A multicenter phase 1b clinical trial found that ACY-1215 is a safe and well tolerated selective HDAC6 inhibitor in combination with lenalidomide and dexamethasone in relapsed or refractory multiple myeloma^16^. However, its efficacy and mechanisms in esophageal cancer remain unclear.

In this study, we evaluated the efficacy and the potential mechanisms of ACY-1215 in ESCC. We found that HDAC6 acted as an oncogene in ESCC. ACY-1215 inhibited proliferation in ESCC, and caused G2/M phase arrest and apoptosis. We integrated the epigenetic characters of HDAC6 and a cancer-related signaling pathway to explore the potential mechanisms of these occurrences. Intriguingly, we further provided evidence that ACY-1215 triggered cell cycle arrest and apoptosis by directly affecting the miR-30d/PI3K/AKT/mTOR and ERK pathways.

## Results

### The high expression of HDAC6 was associated with poor prognosis

**Fig. 1:**
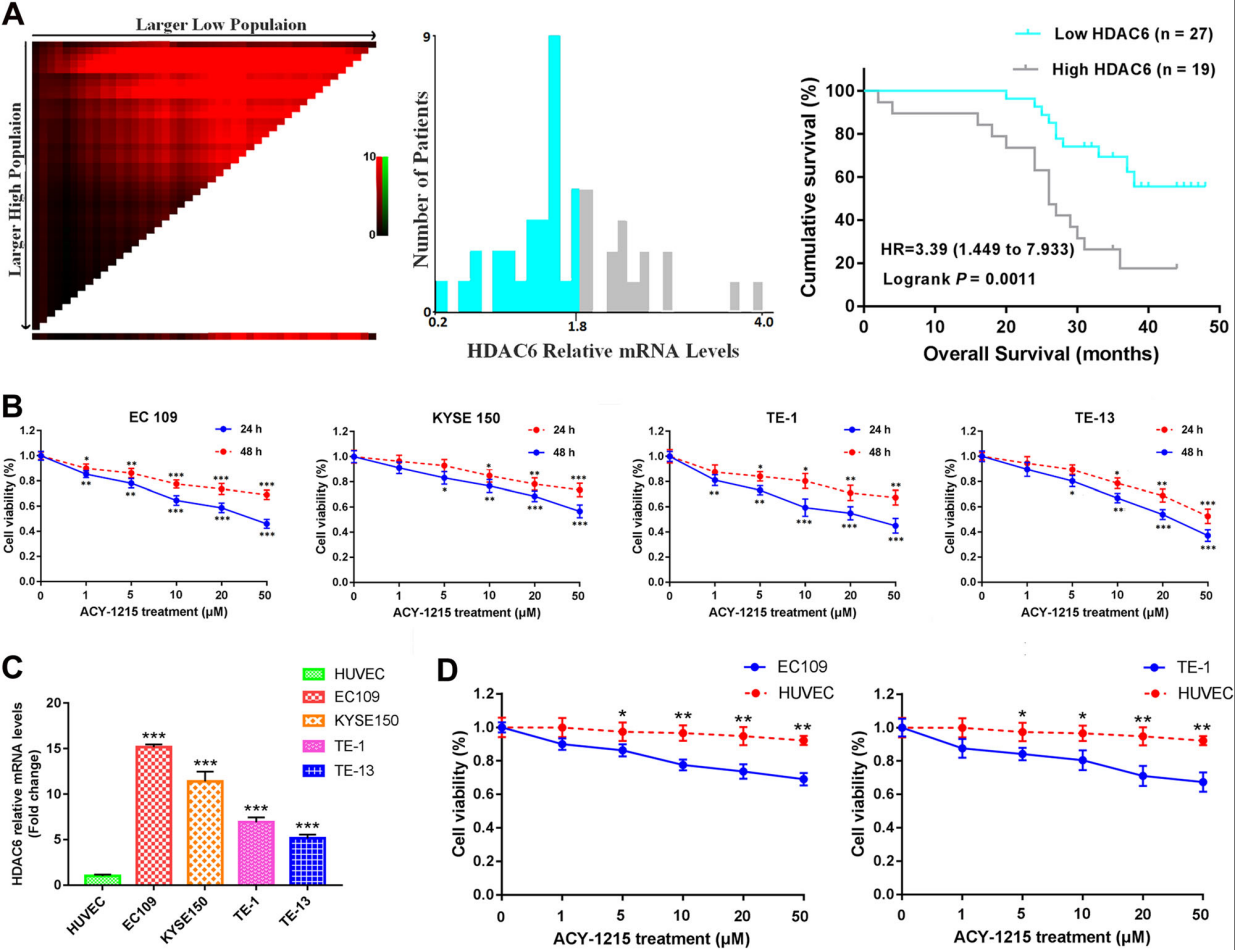
The high expression of HDAC6 was associated with poor prognosis in ESCC specimens, and ACY-1215-inhibited tumor cell proliferation. **a** The optimal cutoff values for HDAC6-related mRNA levels were determined and Kaplan–Meier survival curves for patients with ESCC by HDAC6-relatived mRNA levels. **b** ACY-1215 treatment significantly inhibited cellular proliferation that was dose- and time-dependent manner in EC109 (IC50: 46 μM), KYSE150 (IC50: 57 μM), TE-1 (IC50: 45 μM) and TE-13 (IC50: 37 μM) cells. **c** The relative levels of HDAC6 expression were higher in all ESCC cell lines than that of the HUVEC cell. **d** ACY-1215 inhibited cellular proliferation in HUVEC is significantly less than EC109 and TE-1 cells (24 h). The results are shown as the mean ± SD. *p < 0.05; **p < 0.01; ***p < 0.001

The mechanism of the relationship of HDAC6 with ESCC has not yet been revealed. Therefore, we first used quantitative real-time PCR analysis to identify the expression of HDAC6 in 46 human ESCC specimens. The median follow-up was 31 months (range, 2–48 months). According to the optimal cutoff analyses, the HDAC6-related mRNA levels were classified into low expression (≤1.85) and high expression (>1.85). The high expression of HDAC6 was associated with poor prognosis compared with low expression (*p* = 0.0011, Fig. 1a).

### ACY-1215 inhibited cellular proliferation and caused G2/M arrest and apoptosis

To investigate the antitumor effect of HDAC6 inhibitor in ESCC, we used ACY-1215 treatment against cellular proliferation in EC109, KYSE150, TE-1, and TE-13 cells. The MTT assay revealed that ACY-1215 inhibited cellular proliferation in a dose- and time-dependent manner (Fig. 1b). We also used quantitative real-time PCR analysis to compare the expression of HDAC6 in these ESCC cells with normal HUVEC cell. We found that the relative levels of HDAC6 expression were higher in all ESCC cell lines than that of the HUVEC cell (Fig. 1c). Moreover, we used ACY-1215 treatment against cellular proliferation in the HUVEC cell. The MTT assay revealed that ACY-1215 inhibited cellular proliferation in HUVEC is significantly less than ESCC cells (Fig. 1d).

**Fig. 2:**
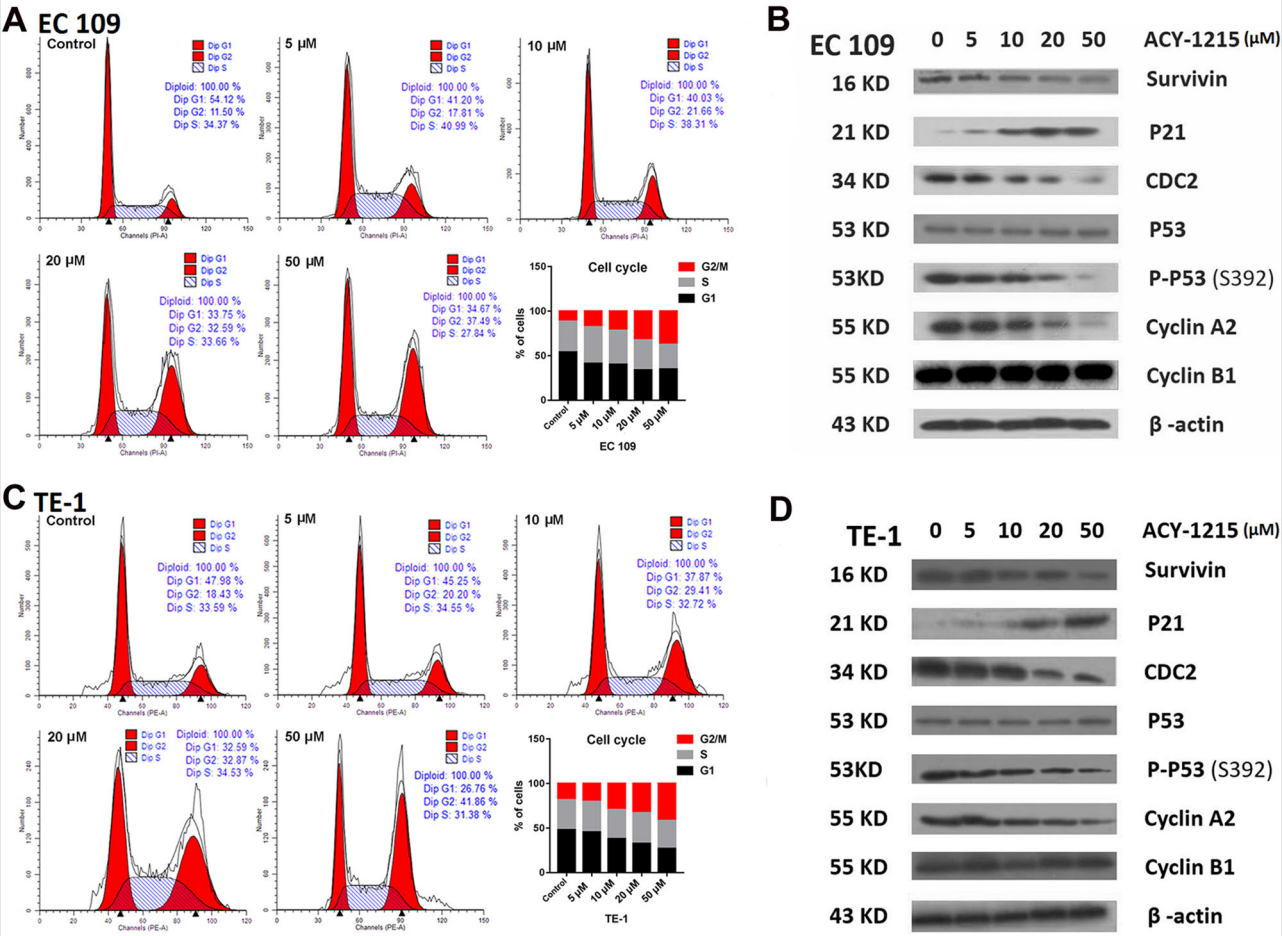
ACY-1215 treatment induced G2/M-phase cell cycle arrest. **a**, **c** ESCC cell lines were treated with ACY-1215 for 48 h. Cells were then stained with propidium iodide, and analyzed by LSR II flow cytometer. ACY-1215 caused a significant increase in the G2/M phase and a decrease in the G1 phase, occurred in a dose-dependent manner. **b**, **d** Western blot analysis of G2/M phase cell cycle regulatory-proteins for 48 h

To clarify the mechanism of ACY-1215 in suppression of tumor cell proliferation, we examined the cell cycle progression and apoptosis in response to varying doses. We observed that G2/M arrest, with an accumulation of cells in the G2/M phase and a decrease in the G1 phase, occurred in a dose-dependent manner (Fig. 2a, c). The G2/M cell cycle-regulatory proteins, such as survivin, CDC2, P-P53, and cyclin A2, all declined in response to ACY-1215 treatment while P21 protein expression increased (Fig. 2b, d).

**Fig. 3:**
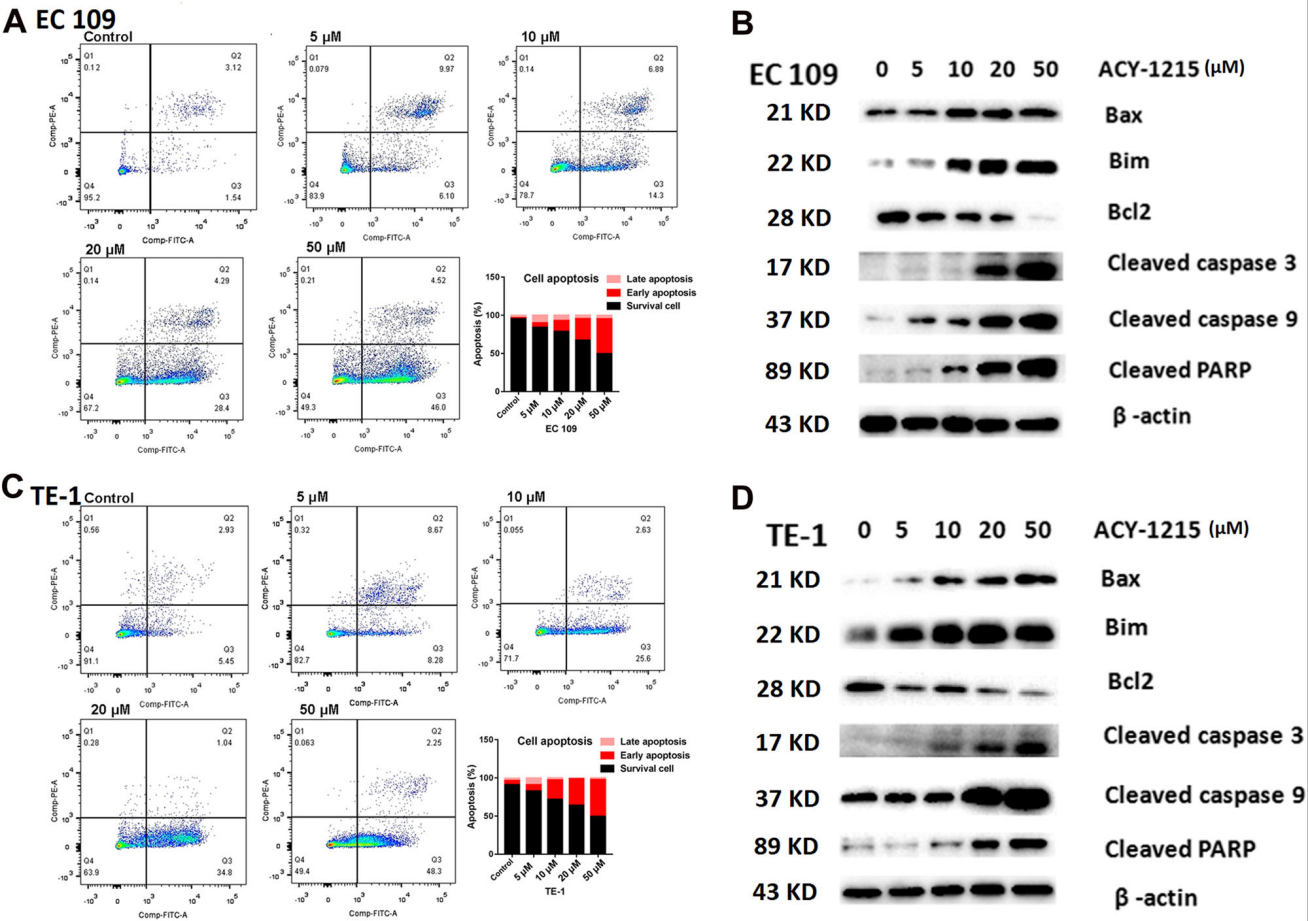
ACY-1215 treatment induced tumor cell apoptosis. **a**, **c** ESCC cell lines were treated with ACY-1215 for 48 h. ACY-1215 caused a significant increase the proportion of apoptosis cells in a dose-dependent manner. **b**, **d** Western blot analysis of apoptosis regulatory-proteins for 48 h

We next evaluated the effect of ACY-1215 on apoptosis and found that the proportion of apoptosis cells significantly increased in a dose-dependent manner (Fig. 3a, c). To identify the apoptosis-regulatory proteins that could mediate this effect, we used western blot analysis to screen the altered proteins after ACY-1215 treatment. This treatment increased the expression of Bax, Bim, cleaved caspase 3 and 9, and cleaved PARP proteins, and decreased Bcl2 protein expression (Fig. 3b, d).

### ACY-1215 inhibited PI3K/AKT/mTOR and ERK signaling and increased intranuclear histone expression

**Fig. 4:**
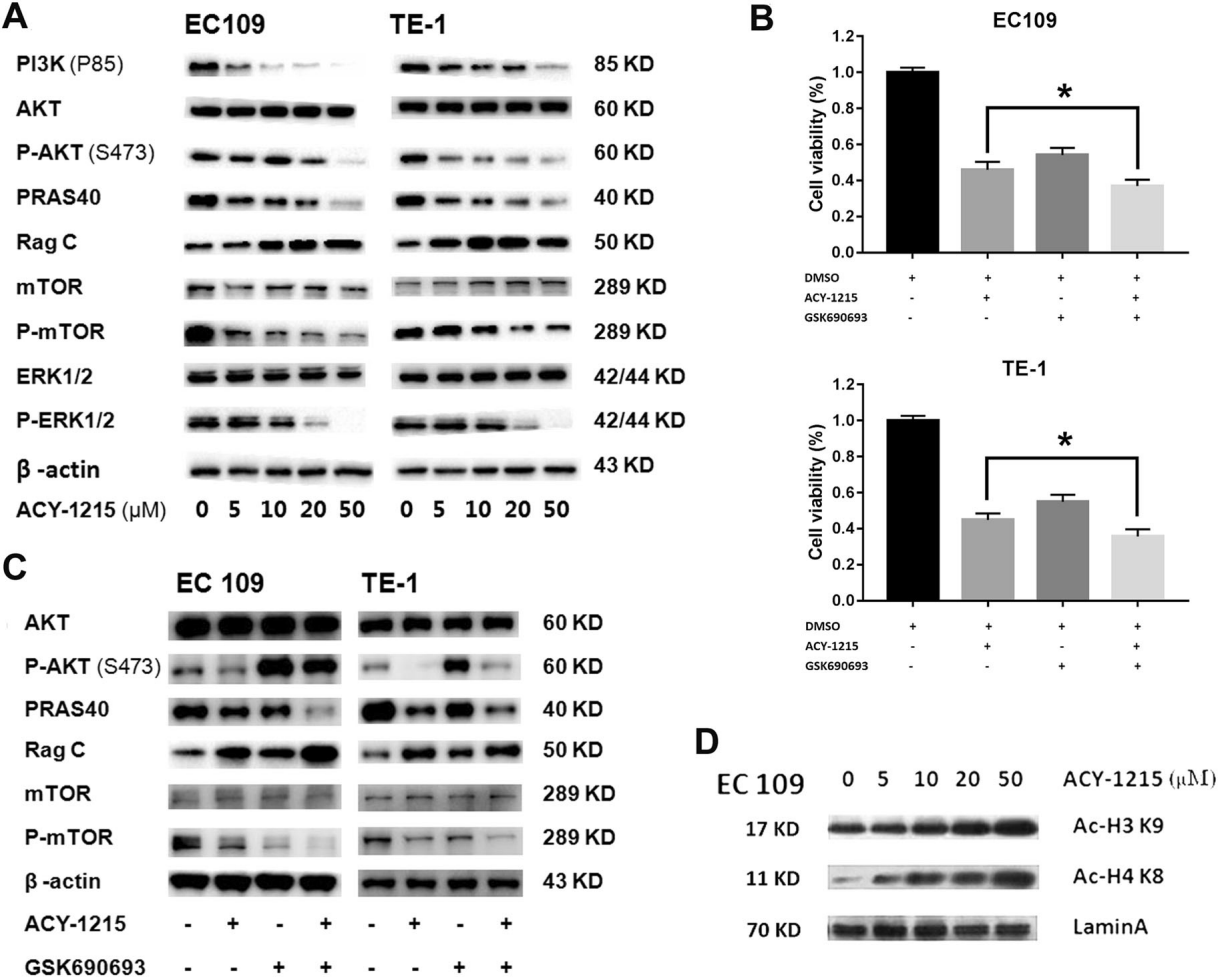
ACY-1215 inhibited PI3K/AKT/mTOR and ERK signaling and increased intranuclear histone expression. **a** ACY-1215 treatment inhibited PI3K/AKT/mTOR and ERK signaling protein levels in ESCC EC109 and TE-1 cell lines for 48 h. **b** GSK690693 (10 μM) combined with ACY-1215 (50 μM) treatment further enhanced the ACY-1215-inhibited cell proliferation (48 h). **c** GSK690693 (10 μM) increased P-AKT due to blocking a negative feedback loop downstream of AKT (PRAS40, Rag C and P-mTOR) for 48 h. **d** ACY-1215 treatment increased intranuclear histones (Ac-H3K9 and Ac-H4K8) levels in ESCC cell lines for 48 h

To better understand the mechanism by which ACY-1215 caused tumor cell cycle arrest and apoptosis, western blot was carried out to explore the effect of ACY-1215 on its other targets. We demonstrated that ACY-1215 effectively reduced PI3K, P-AKT (S473), PRAS40, P-mTOR, and P-ERK1/2 protein levels and increased Rag C protein levels (Fig. 4a). In addition, using GSK690693 (a pan-Akt inhibitor) combined with ACY-1215 treatment the ESCC cells, we detected that co-treatment with GSK690693 could further enhance the ACY-1215-inhibited cell proliferation (Fig. 4b). GSK690693 increased P-AKT due to blocking a negative feedback loop downstream of AKT (PRAS40, Rag C, and P-mTOR) (Fig. 4c).

As HDAC6 plays an important epigenetic role in modulating the transcription of many genes, we extracted nuclear protein and explored the expression of intranuclear histones by western blot analysis. We found that the levels of Ac-H3K9 and Ac-H4K8 were increased after ACY-1215 treatment (Fig. 4d).

### ACY-1215 promoted miR-30d expression and miR-30d directly downregulated PI3K

**Fig. 5:**
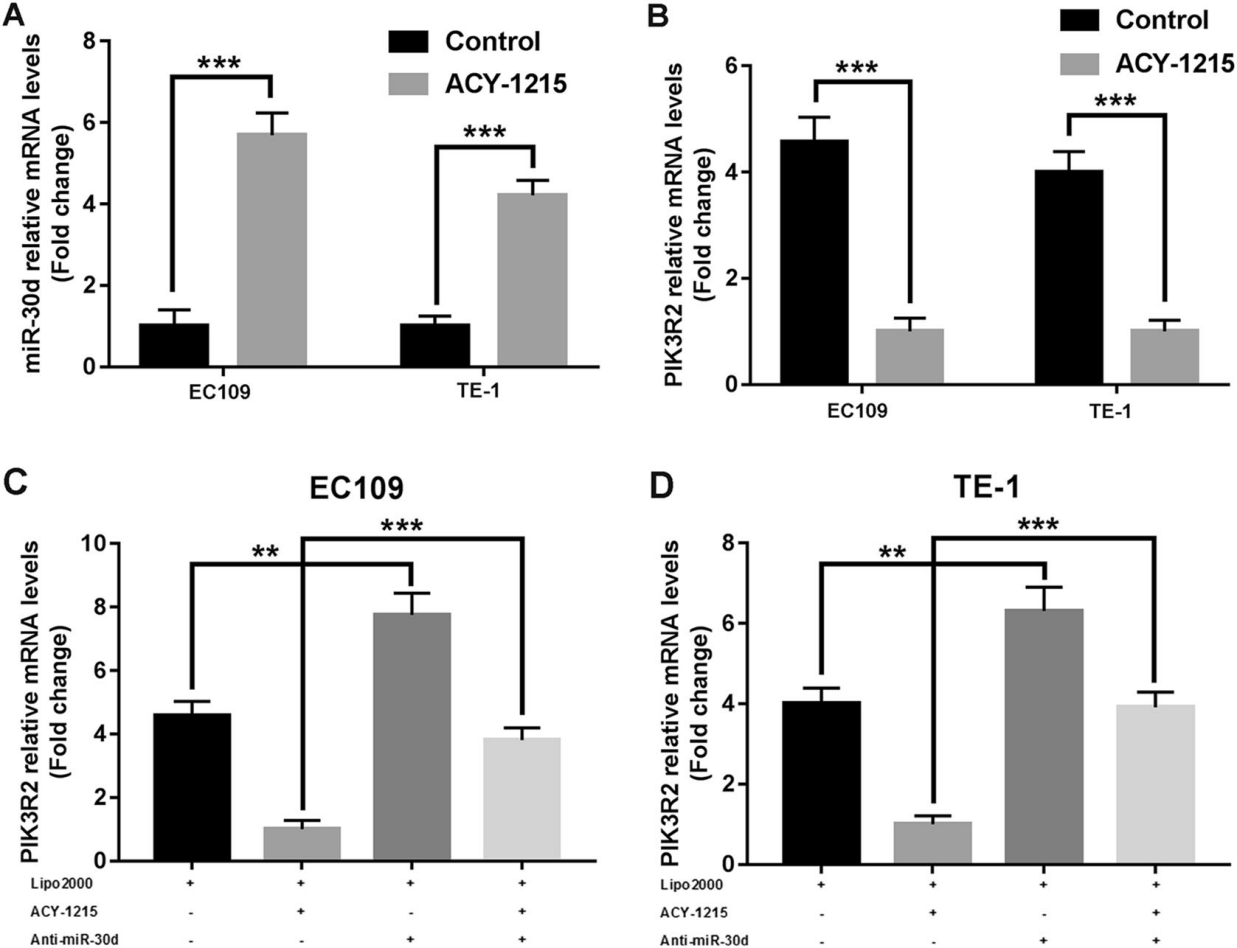
ACY-1215 promoted miR-30d expression, and miR-30d directly downregulated PI3K. **a** ACY-1215 treatment significant increased miR-30d expression in EC109 and TE-1 cells for 48 h (50 μM). **b** ACY-1215 treatment significant reduced PIK3R2 expression in EC109 and TE-1 cells for 48 h (50 μM). **c**, **d** Cells were treated with Lipofectamine 2000, 50 μM ACY-1215, and/or 50 nM miR-30d inhibitor for 48 h. The quantitative real-time PCR analysis demonstrated that PIK3R2 expression significantly increased after cells were transfected with miR-30d inhibitor, particularly in the ACY-1215 treatment group. The results are shown as the mean ± SD. **p < 0.01; ***p < 0.001

Having confirmed that ACY-1215 was correlated with epigenetic effects, we used miRNA microarray assay to investigate those miRNAs whose expression was upgraded by ACY-1215 treatment. A total of 46 candidate miRNAs, with at least an 8-fold rise in signal levels, were found. Then, we used three publicly available prediction algorithms, Targetscan (http://www.targetscan.org), Pictar (http://pictar.mdc-berlin.de), and miRanda (http://www.microrna.org), to explore potential miRNAs involved in the PI3K/AKT signaling pathway. We found that miR-30d directly downregulated PI3K regulatory subunit 2 (PIK3R2). To validate the results, we used quantitative real-time PCR analysis to demonstrate that miR-30d was significantly overexpressed after ACY-1215 treatment, while PIK3R2 expression decreased (Fig. 5a, b). Furthermore, we found that when ESCC cells were transfected with miR-30d inhibitor, PIK3R2 expression significantly increased, particularly in the ACY-1215 treatment group, further validating that PIK3R2 is a direct target gene of miR-30d (Fig. 5c, d).

### Anti-miR-30d partially restored the G2/M arrest and apoptosis and AKT signaling caused by ACY-1215 treatment

**Fig. 6:**
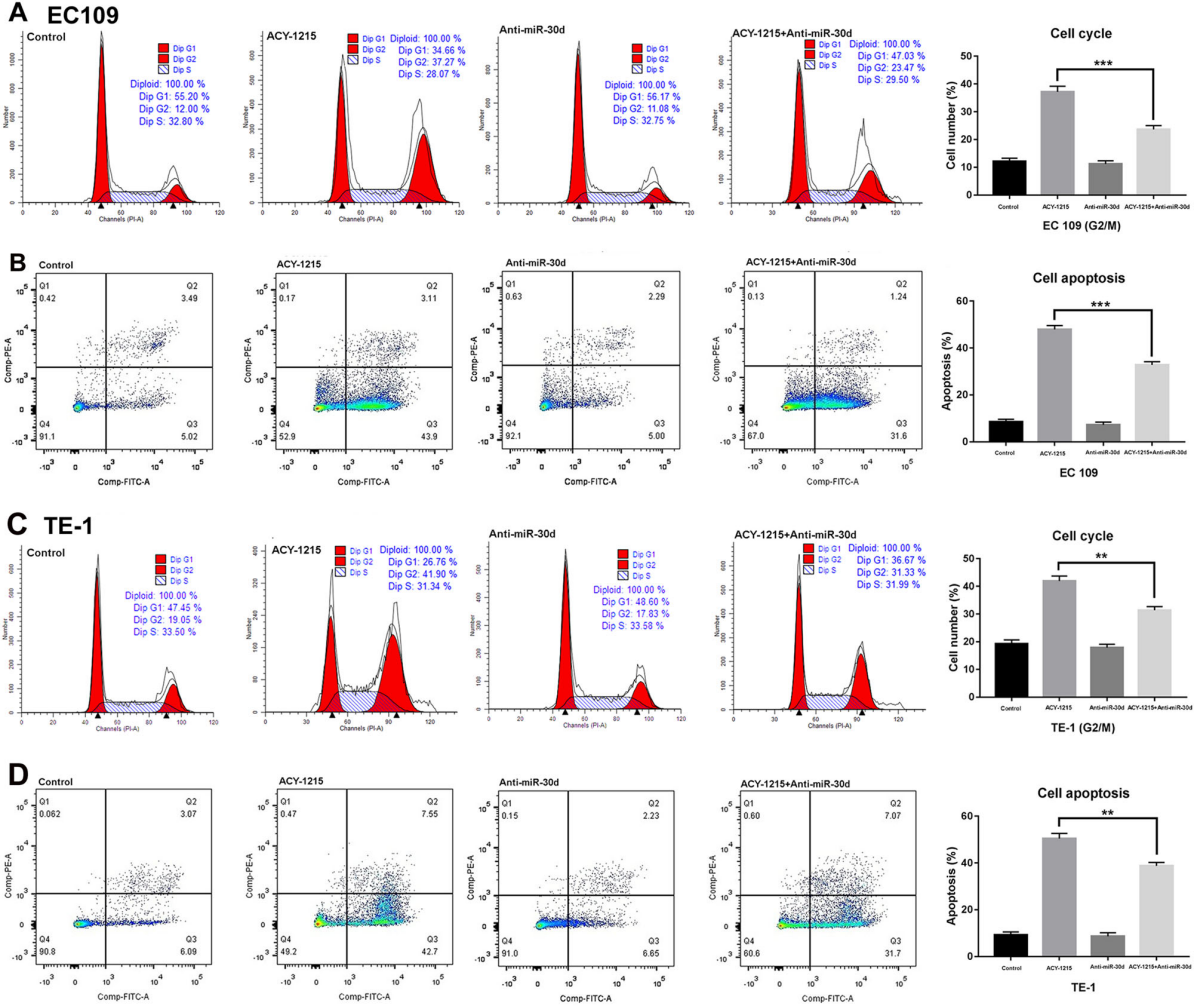
Anti-miR-30d partially restored the G2/M arrest and apoptosis and AKT signaling caused by ACY-1215 treatment. **a**, **c** The ACY-1215 (50 μM) induced G2/M phase cell cycle arrest was partially rescued by anti-miR-30d (50 nM). Cells were treated with Lipofectamine 2000, 50 μM ACY-1215, and/or 50 nM miR-30d inhibitor for 48 h. **b**, **d** The ACY-1215 (50 μM) induced apoptosis was partially rescued by anti-miR-30d (50 nM). Cells were treated with Lipofectamine 2000, 50 μM ACY-1215, and/or 50 nM miR-30d inhibitor for 48 h. The results are shown as the mean ± SD. **p < 0.01; ***p < 0.001

**Fig. 7:**
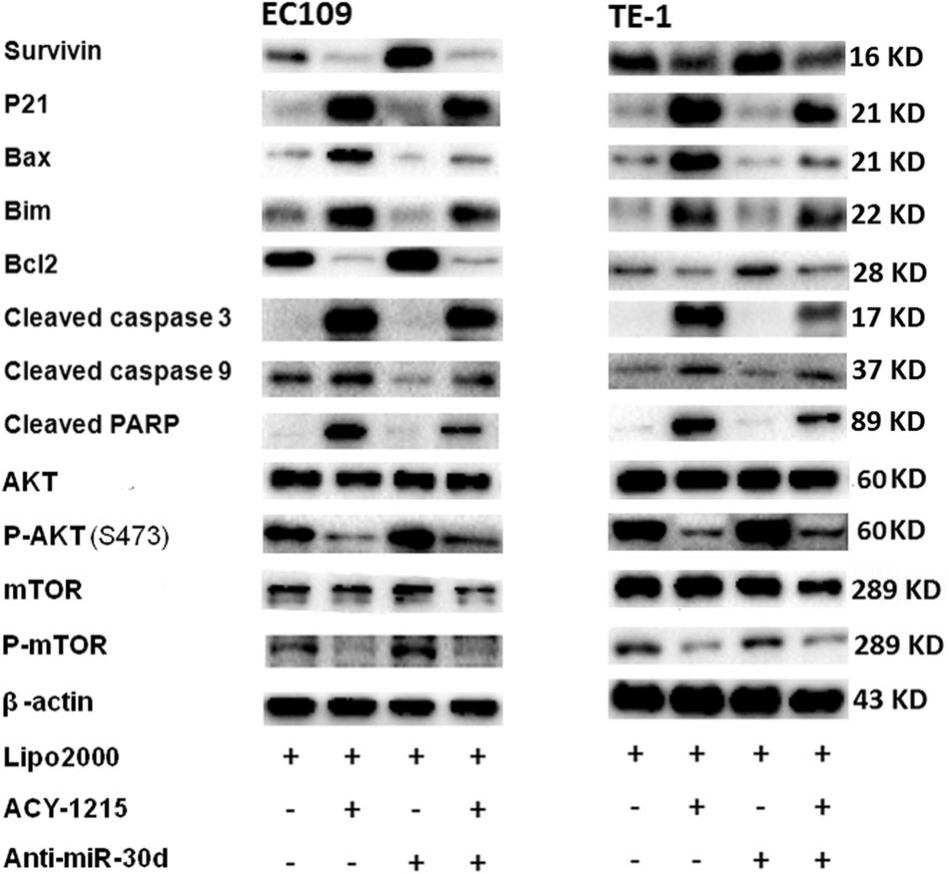
Cells were treated with Lipofectamine 2000, 50 μM ACY-1215, and/or 50 nM miR-30d inhibitor for 48 h. Western blot analysis demonstrated that the ACY-1215 induced changes in the expression of cell cycle-regulatory and apoptosis-regulatory proteins and AKT signaling proteins were also partially reversed by anti-miR-30d

To clarify the underlying functional mechanisms of miR-30d in ESCC after ACY-1215 treatment, we transfected miR-30d inhibitor into the tumor cells and found that the ACY-1215-induced G2/M arrest and apoptosis were partially rescued by anti-miR-30d (Fig. 6a–d). Moreover, Western blot analysis demonstrated that the ACY-1215-induced changes in the expressions of cell cycle-regulatory and apoptosis-regulatory proteins and AKT signaling proteins were also partially reversed by anti-miR-30d (Fig. 7). In summary, these data validated the conclusion that ACY-1215 suppresses proliferation and promotes apoptosis in ESCC by upregulating miR-30d and inhibiting PI3K/AKT/mTOR signaling pathways.

### ACY-1215 inhibited tumor growth in a mouse xenograft model

**Fig. 8:**
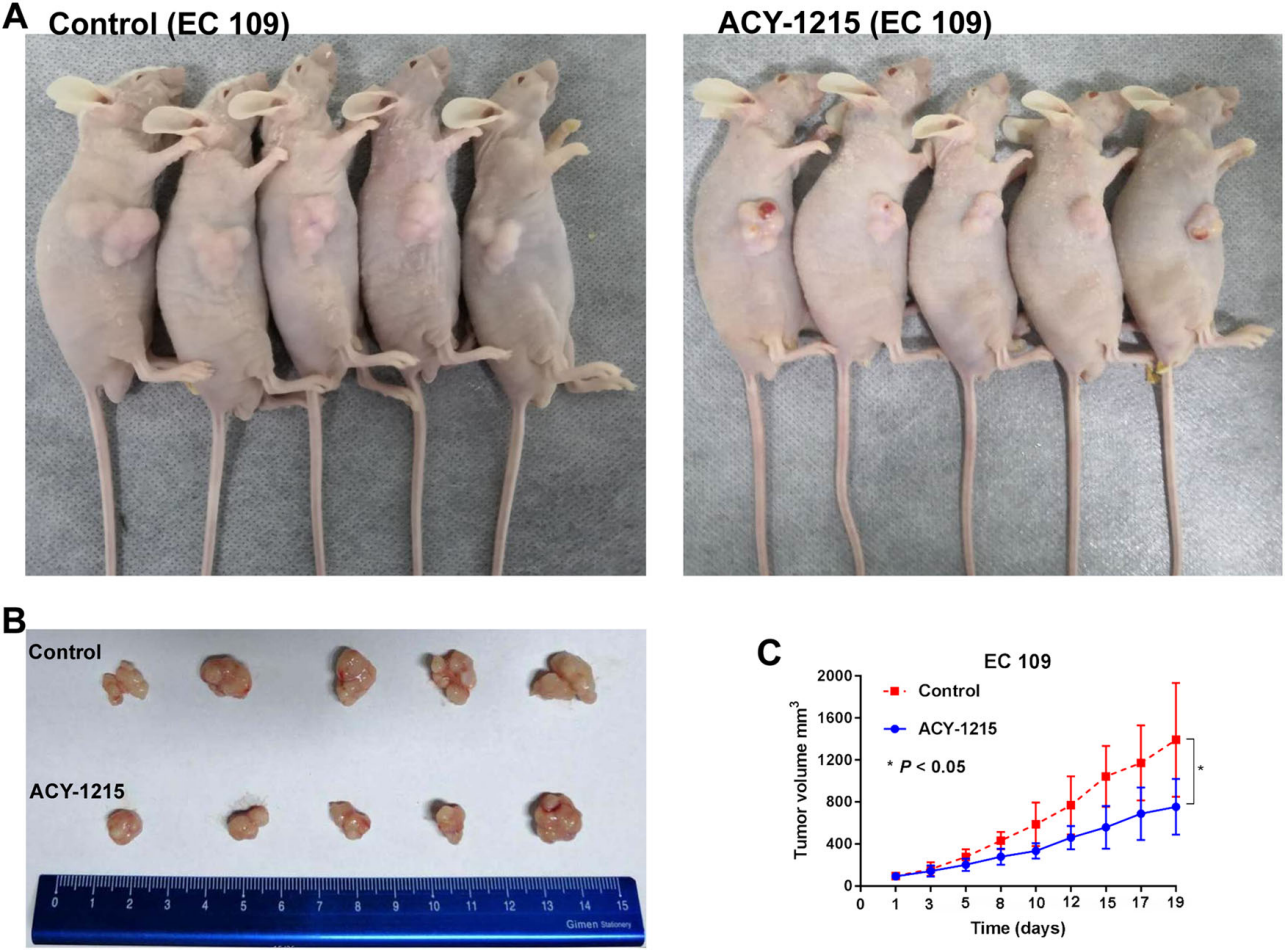
ACY-1215 treatment led to significant tumor growth slowed in vivo. **a**, **b** EC109 cells were injected subcutaneously into the right side of each BALB/c male nude mice. After three cycle of therapy, animals were sacrificed and tumors were excised. **c** Tumor diameters were measured after therapy 3 times every week, and tumor volumes were calculated. Values are mean ± SD. *p < 0.05

To further validate the effects of ACY-1215 in vivo, EC109 cells were injected subcutaneously into the BALB/c nude mice. Ten days after injection, mice were divided into control group and ACY-1215 treatment group, and the latter group was treated with ACY-1215 50 mg/kg via intraperitoneally. Treatment was well tolerated. Following three cycles of therapy, the ACY-1215 treatment group led to a statistically significant tumor growth slower than those in the control group (Fig. 8a–c). These findingssuggested that ACY-1215 inhibited tumor growth in vivo.

## Discussion

Several new observations in this study may provide important insights into the efficacy of ACY-1215 and its underlying mechanisms in ESCC. First of all, we identified a relationship between HDAC6 and ESCC: the high expression of HDAC6 was associated with poor prognosis. Secondly, the HDAC6-selective inhibitor ACY-1215 inhibited cellular proliferation and caused G2/M arrest and apoptosis via PI3K/AKT/mTOR and ERK pathways. Finally, we confirmed that ACY-1215 was correlated with epigenetic modulation in ESCC. We found that the levels of Ac-H3K9, and Ac-H4K8 protein expression were increased after ACY-1215 treatment, and miR-30d directly targeted PIK3R2 to inhibit PI3K/AKT/mTOR signaling pathways.

Recently, it has been identified that HDAC6 is overexpressed in several malignancies^17^. In acute lymphoblastic leukemia, oral squamous cell carcinoma, ovarian cancer, and hepatocellular carcinoma, HDAC6 acts as a tumor inducer: its overexpression is correlated with a more advanced stage and increased tumor aggressiveness^18–21^. However, in cutaneous T-cell lymphoma and chronic lymphocytic leukemia, HDAC6 overexpression is correlated with longer survival^22,23^. Meanwhile, in breast cancer, HDAC6 plays a dual role, and its expression is regulated by estrogen^24,25^. However, the expression of HDAC6 in esophageal cancer previously remained unknown. We found that HDAC6 acts as an oncogene in ESCC, which indicates that HDAC6 may also serve as a target for drug development to treat ESCC.

ACY-1215 is a first-in-class potent and selective HDAC6 inhibitor. It has demonstrated antitumor effects, either as a single treatment or in combination with other agents, by a range of mechanisms^6,9^. In multiple myeloma, ACY-1215 has a specific inhibitory effect on HDAC6 activity: even a very low dose can induce potent acetylation of α-tubulin, but triggering the acetylation of lysine on histone H3 and histone H4 needs higher doses. ACY-1215 in combination with bortezomib can induce apoptosis by activation of endoplasmic reticulum (ER) stress and unfolded protein response in two xenograft mouse models^10^. In non-Hodgkin lymphoma cells, ACY-1215 acts synergistically with carfilzomib through multiple stress-related mechanisms, causing increases in DNA damage, G2/M arrest, and inducing mitochondrial injury and apoptosis^12^. In glioblastoma, tumor cell growth is significantly inhibited by ACY-1215 through transforming growth factor β receptor signaling, which induces SMAD2 phosphorylation and increased P21 expression^13^. In BRAF-mutant melanoma cells, ACY-1215 sensitizes vemurafenib-induced cell proliferation inhibition and apoptosis through induction of ER stress and inactivation of ERK^14^.

In ESCC, we demonstrated that ACY-1215 inhibited cellular proliferation by inducing G2/M arrest and apoptosis. Regulatory proteins involved in G2/M transition and dysregulated in ESCC were variously downregulated (survivin, cdc2, P-P53, and cyclin A2) and upregulated (P21) by ACY-1215 treatment. Survivin is also an inhibitor of apoptosis, wherein survivin inhibition amplifies caspase activation^24^. In addition to increasing apoptosis through the commonly activated caspase pathway, we also identified that ACY-1215 increased Bax and Bim protein expression and reduced Bcl2 protein levels. To better understand the mechanism by which ACY-1215 caused tumor cell cycle arrest and apoptosis, we demonstrated that ACY-1215 not only effectively inhibited phosphorylation ERK, but also inhibited PI3K/AKT/mTOR signaling pathways. GSK690693 (a pan-Akt inhibitor) combined with ACY-1215 treatment could further enhance the ACY-1215-inhibited cell proliferation. GSK690693 increased P-AKT due to blocking a negative feedback loop downstream of AKT (PRAS40, Rag C, and P-mTOR). PI3K/AKT/mTOR signaling is a classical pathway involved in ECSS initiation and/or progression^25^. A recent study showed that ACY-1215 in combination with bendamustine leads to AKT pathway inactivation in lymphoma cells. The phosphorylation status of AKT, GSK3β, mTOR, 4EBP1, p90RSK, and p70S6 kinase all declined under treatment with ACY-1215 in combination with bendamustine^26^. In this study, we identified that ACY-1215 effectively reduced PI3K, P-AKT (S473), PRAS40, and P-mTOR protein levels in ESCC. Thus, the effective targeting of multiple downstream effectors suggests that ACY-1215 may be an effective anticancer agent for ESCC.

In addition, HDAC6 has important epigenetic characteristics. Several recent studies have demonstrated that miR-22, miR-27b, miR-206, miR-221, miR-433, and miR-548m are involved in the regulation of HDAC6 in cutaneous T-cell lymphoma, large B-cell lymphoma, head and neck squamous cell carcinoma, liver cancer, oral squamous cell carcinoma, and non-Hodgkin B cell lymphomas, respectively^27–32^. However, despite ACY-1215 being a first-in-class potent and selective HDAC6 inhibitor, one study found that only higher doses of ACY-1215 triggered acetylation of lysine on histone H3 and histone H4^10^. In the present study, we found that ACY-1215 not only triggered acetylation of lysine on histone H3K9 and histone H4K8, but also promoted miR-30d expression.

MiR-30d, a member of the miR-30 family, is located in the 8q24.22^33^. It has been implicated in several tumors, playing a role as a tumor suppressor and regulating a variety of target genes, thus inhibiting the initiation and progression of tumors^34–37^. In ESCC, we found that miR-30d directly downregulated PI3K regulatory subunit 2 (PIK3R2), and when ESCC cells were transfected with miR-30d inhibitor, PIK3R2 expression significantly increased. A previous study also identified that miR-30d targeted the 3′-UTR of PIK3R2 to regulate glucose metabolism in skeletal muscle cells^38^. Moreover, we demonstrated here that anti-miR-30d partially reversed the G2/M arrest and apoptosis and AKT signaling caused by ACY-1215 treatment, further indicating that ACY-1215 suppresses proliferation and promotes apoptosis in ESCC through miR-30d/PI3K/AKT/mTOR signaling pathways.

In conclusion, we identified that HDAC6 acts as an oncogene in ESCC. A selective HDAC6 inhibitor, ACY-1215, inhibited proliferation in ESCC, and caused G2/M phase arrest and apoptosis via miR-30d/PI3K/AKT/mTOR and ERK pathways. Altogether, these findings provide underlying molecular and cellular evidence that ACY-1215 may be a promising antitumor agent in ESCC.

## Materials and methods

### Clinical specimens

In total, 46 human ESCC specimens were collected in the Department of Thoracic Surgery, First Affiliated Hospital, College of Medicine, Zhejiang University, between June 2013 and October 2014. Written informed consent was obtained from all patients before the specimens were collected and the study protocol was approved by the institutional ethical review board. Survival time was calculated from the date of diagnosis to the date of death, the last contact, or the cutoff date of July 31, 2017.

### Cell culture and reagents

The human cell lines EC109, KYSE150, TE-1, TE-13, and HUVEC were obtained from the Cell Bank of the Chinese Academy of Sciences (Shanghai, China) and cultured in DMEM medium (Gibco, USA) containing 10% fetal bovine serum. Ricolinostat (ACY-1215) was purchased from Selleck Chemicals (TX, USA) and was dissolved in DMSO to obtain a stock concentration of 100 mM. MiR-30d inhibitor was purchased from RiboBio Company (Guangzhou, China) and was dissolved in RNase-free water to obtain a concentration of 20 µM.

### Quantitative real-time PCR

Total RNA was extracted from tissues or cells using an RNeasy Mini Kit (Qiagen, Germany) and used to synthesize cDNA (Vazyme, USA) according to the standard instructions. MiRNA was extracted from cells using a miRNeasy Mini Kit (Qiagen, Germany) and used to synthesize cDNA (Qiagen, Germany) according to the standard instructions. Quantitative real-time PCR (Vazyme, USA) was performed according to the manufacturer’s instructions. The primer sequences were as follows: human HDAC6 forward: 5′- AAGACCTAATCGTGGGACTGC-3′ and reverse: 5′-ACGTGGTTGAACATGCAATAG-3′; human GAPDH forward: 5′-GTCTCCTCTGACTTCAACAGCG-3′ and reverse: 5′-ACCACCCTGTTGCTGTAGCCAA-3′; miR-30d: 5′-TGTAAACATCCCCGACTGGAAG-3′; U6: 5′-TGCGGGTGCTCGCTTCGGCAGC-3′; human PIK3R2 forward: 5′-AATGCAGCAAGGAATACCTGG-3′ and reverse: 5′-G CTCTCATGGATCTCGGCAA-3′.

### Cell proliferation, cell cycle, and apoptosis assays

Cell proliferation was assessed by 3-(4,5-dimethylthiazol-2-yl)-2,5-diphenyl tetrazolium bromide (MTT) assays, as described in a previous study^39^. Cell cycle analysis was performed as described previously^40^. The samples were analyzed using an LSR II flow cytometer (BD Biosciences) and ModFitLT software (ME, USA). Apoptosis was measured by Annexin V/propidium iodide staining. The results were analyzed using Flowjo software (CT, USA).

### Western blotting assays and antibodies

Cells were lyzed in ice-cold radioimmunoprecipitation assay lysis buffer (Beyotime Institute of Biotechnology) to extract the total protein. The nuclear protein was extracted using a Thermo Scientific NE-PER kit (Thermo Fisher Scientific) according to the standard instructions. Western blotting was performed as previously described^40^. The following antibodies were purchased from Cell Signaling Technology (Danvers, MA): Survivin (dilution at 1:1000), P21 (1:1000), CDC2 (1:1000), P53 (1:1000), P-P53 (1:1000), CyclinA2 (1:1000), CyclinB1 (1:1000), Bax (1:1000), Bim (1:1000), Bcl2 (1:1000), Cleaved caspase3 (1:1000), Cleaved caspase 9 (1:1000), Cleaved PARP (1:1000), PI3K (1:1000), AKT (1:1000), P-AKT (1:1000), PRAS40 (1:1000), Rag C (1:1000), mTOR (1:1000), P-mTOR (1:1000), ERK1/2 (1:1000), P-ERK1/2 (1:1000). Ac-H3K9 (1:1000), Ac-H4K8 (1:1000), LaminA70 (1:5000), and β-action (1:5000) antibodies were purchased from Abcam (Cambridge, MA).

### MiRNA microarray assay

Microarray assay was performed using a service provider (LC Sciences). The hybridization reaction was performed overnight on a µParaflo microfluidic chip using a micro-circulation pump (Atactic Technologies). The detection probes were synthesized by photogenerated reagent chemistry. Hybridization used 100 μL of 6× SSPE buffer (0.90 M NaCl, 60 mM Na_2_HPO_4_, 6 mM EDTA, pH 6.8) containing 25% formamide at 34 °C. Fluorescence images were collected using a laser scanner (GenePix 4000B, Molecular Devices) and digitized using Array-Pro image analysis software (Media Cybernetics).

### Oligonucleotide transfection

Cells were transfected in 6-well plates with a miR-30d inhibitor, using Lipofectamine 2000 reagent (Invitrogen, Carlsbad, CA, USA). The miR-30d inhibitor and Lipofectamine 2000 reagent were diluted in Opti-MEM Medium (Gibco, New York, USA). The final volume of Lipofectamine 2000 reagent per well was 5 μL, and the final miR-30d inhibitor concentration was 50 nM. After transfection for 48 h, cells were harvested for the assays.

### In vivo studies

Animals were housed and maintained under specific pathogen-free conditions. EC109 cells (2 × 10^6^) were injected subcutaneously into the right side of each 5–6-week-old BALB/c male nude mice (Animal Center of the Chinese Academy of Science, Shanghai, China). Treatment was initiated when tumors reached a volume of 50–150 mm^3^. Tumor volume was calculated as the largest length × width^2^ × 0.5 and tumor diameter was measured three times every week. Mice were divided into control group and ACY-1215 treatment group. Each group contained five animals. ACY-1215 was diluted in 0.9% saline and was intraperitoneally administrated with a daily dose of 50 mg/kg for days 1–5, 8–12, and 15–19. All procedures were approved by the Institutional Animal Care and Use Committee.

### Statistical analysis

The optimal cutoff values for HDAC6-related mRNA levels were determined using X-Tile software and by the minimal *p*-value approach. We used the Kaplan–Meier method to estimate overall survival curves, and evaluated statistical differences using the log-rank test. The other data were analyzed by the two-tailed Student’s *t* test and presented as the mean ± standard deviation. Statistical analyses and graphics-creation were performed using GraphPad Prism 7.0 (GraphPad, Inc, CA, USA). For all of the analyses, *p* < 0.05 was considered to be statistically significant.
